# Prolonged grief and posttraumatic stress disorder following the loss of a significant other: An investigation of cognitive and behavioural differences

**DOI:** 10.1371/journal.pone.0248852

**Published:** 2021-04-01

**Authors:** Kirsten V. Smith, Anke Ehlers

**Affiliations:** 1 Department of Experimental Psychology, University of Oxford, Oxford, United Kingdom; 2 Oxford Health NHS Foundation Trust, Oxford, United Kingdom; 3 The Loss Foundation, London, United Kingdom; Koc University School of Medicine, TURKEY

## Abstract

**Background:**

Cognitive behavioural correlates to bereavement-related mental health problems such a Prolonged Grief Disorder (PGD) and Posttraumatic Stress Disorder (PTSD) are of theoretical and clinical importance.

**Methods:**

Individuals bereaved at least six months (N = 647) completed measures of loss-related cognitions and behaviours (i.e., loss-related memory characteristics, negative appraisals, coping strategies, grief resilience, and perceived social disconnection) and measures of PGD and PTSD symptoms. Individuals were assigned to one of four groups depending on probable clinical diagnoses (No-PGD/PTSD, PTSD, PGD, PGD+PTSD).

**Results:**

Results indicated that higher loss-related memory characteristics and lower grief resilience increased the likelihood of a clinical problem. The PGD and PGD+PTSD groups reported significantly higher loss-related memory characteristics and appraisals compared to the PTSD group. Social disconnection increased the likelihood of comorbid PGD+PTSD in comparison to any other group.

**Conclusions:**

Results indicate cognitive differences between loss-related cognitions, memory characteristics and coping strategies between PGD and PTSD, and points to distinct cognitive correlates to psychopathology following loss.

## Introduction

Following bereavement, a minority of individuals develop mental health problems. Some individuals develop one or both of the stress-response syndromes posttraumatic stress disorder (PTSD) and prolonged grief disorder (PGD) [1,2]. PTSD is characterised by symptoms of hyperarousal, avoidance and intrusive trauma-related memories. PGD is characterised by intense separation distress, difficulties accepting the loss, and moving on without the lost person, which causes significant distress and disability. Several theoretical models of PGD [3–6] and PTSD [7] implicate cognitive processes in the development and maintenance of distressing symptoms, in particular negative appraisals of the loss or its consequences (e.g., about the meaninglessness of life without the deceased, threatening interpretations of grief reactions) [8], unhelpful coping strategies (e.g., avoidance, rumination) [9,10] and characteristics of loss-related memories (e.g. intrusiveness, type of triggers, “here and now” quality, predominance of negative reactions and visceral consequences). The question of whether there are differential cognitive correlates to each of these disorders is of clinical and theoretical relevance, as it would allow therapists and researchers to home in on the characteristic maintenance processes for each disorder.

Given the recent uncertainty regarding the diagnostic criteria for PGD, studies have typically sought predictors of importance by associating variables with PGD symptom severity [9–11], and a few studies have examined cognitive factors that correlate with PTSD following bereavement [12–14].

A number of recent studies have sought to investigate correlates of PGD and PTSD symptom profiles after loss by identifying distinct groups of individuals suffering from one of the disorders [15–21] using the proposed symptoms for PGD [22] or the DSM criteria for PTSD [23]. One such study used latent class analysis (LCA) to investigate PGD and PTSD symptom profiles and their correlates in a sample of adult refugees who had lost a loved one [17]. They found four classes of individuals: resilient, PGD, PTSD, and combined PGD+PTSD. Interestingly, they found evidence of differential correlates of each clinical class in comparison to the resilient class, suggesting specific etiological pathways. Membership in the PGD+PTSD group was predicted by exposure to traumatic loss, detention, and abuse traumas. The PGD group were more likely to have reported adaptation difficulties since relocation than the resilient group, and the PTSD group had experienced significantly more difficulties related to loss of culture and support. The authors highlight the importance of understanding the dual impact of loss and trauma on mental health characterised by high PGD and PTSD symptoms. This study only used the resilient class as the comparison group and as such it is unclear what, if any, differences could be observed between the clinical classes when compared to each other. In another LCA analysis with bereaved individuals exposed to armed conflict the same comparison groups were extracted [19]. Results indicated that, compared to the resilient class, the PGD class were more likely to have lost a close relative and been exposed to more assaultive trauma while the PTSD class was predicted by less perceived social support. The combined PGD+PTSD class were more likely to have lost a close relative, experienced more accidental and assaultive trauma, and feel less socially supported. Comparing the PGD and PTSD alone classes saw the PGD group experience more losses of close relatives and less time since bereavement. However, the combined classes were not compared with the PGD or PTSD alone classes.

Very few studies have examined the differences in appraisals between different symptom profiles after loss [18,21]. Mccallum & Bryant (2018) investigated negative beliefs about the self, world, and self-blame in the prediction of PGD and PTSD using LCA. They found three classes: resilient, PGD alone, and PGD+PTSD. Compared with the resilient class, the PGD alone and comorbid PGD+PTSD classes were predicted by negative self-appraisals, but they did not differ from each other.

PGD and PTSD share similarities (e.g. both are triggered by a stressful life event and are thought to result from a failure of memory integration) [24]. However, there are also clear clinical differences such as the range of emotions prompted by the disorder (i.e. fear, shame for PTSD and yearning for PGD; with guilt, sadness and anger common in both) and the presence or absence of hyperarousal (i.e. common in PTSD, but not PGD) [25]. Therefore, if distinct cognitive correlates of each disorder were identified, this would increase our understanding of the disorders and allow for tailored interventions. While latent class approaches are useful in determining symptom profiles in place of diagnostic criteria for PGD and PTSD, they are limited in that comparisons of class correlates can only be made on the symptom profiles that emerge.

In this study, we aimed to compare four groups following bereavement: NoPGD/PTSD, PTSD, PGD and PGD+PTSD on loss-related cognitive and behavioural factors (i.e., memory characteristics, appraisals, coping strategies, social disconnection and grief resilience). In keeping with the methods that will be most readily utilised in clinical settings, and to ensure direct comparison of these groups of interest, membership of the clinical groups was determined by probable diagnoses of PGD and PTSD on the basis of questionnaire measures. When planning the study, we decided to use the PGD-2009 criterion proposed by Prigerson et al. [22], which was expected to be adopted in the most recent revision of the International Classification of Diseases (ICD-11), as well the DSM-5 criteria for PTSD [23]. However, ICD-11 has recently adopted a shorter two item criterion [26] for PGD. Proponents of this new criterion argue that with fewer symptoms the new conceptualisation will be easier for clinicians to memorise and apply to diverse contexts around the world. However, the new ICD-11 PGD criteria have been criticised for conflating empirical support for previously proposed criteria for PGD [22] and persistent complex bereavement disorder (PCBD; DSM-5) as confirming the validity of the new ICD-11 PGD symptom criteria [27]. Recently, it was argued that these criteria risk overdiagnosis [27], citing a community-based study that found prevalence of probable ICD-11 PGD (18.0%) was substantially higher than persistent complex bereavement disorder (PCBD) according to DSM-5 [23] (6.4%) [28]. Given the current lack of empirical support for the new ICD-11 PGD criterion we decided to retain the PGD-2009 criterion [22] and compare the pattern of results with those for the criteria for PCBD to further validate our findings.

Due to the partial overlap between grief and trauma-related stress symptoms, and the cognitive theories proposed to explain them [3–5,7], we hypothesised that all three clinical groups (PTSD only, PGD only, PGD+PTSD) would score significantly higher on the loss-related cognitive measures than those without PGD or PTSD (i.e., the non-clinical bereaved group). As the measures were specifically designed to predict grief reactions, it was expected that individuals suffering from PGD would score higher on our chosen measures than those suffering PTSD, and that suffering from both disorders would result in the highest scores.

We further investigated whether the cognitive factors were able to significantly distinguish between groups after examining the relative contribution of moderator variables such as demographics and loss characteristics, as well as pre-loss individual differences previously shown to be associated with grief severity such as attachment style [29] and dependency [30,31].

## Methods

### Participants

Participants were 647 adults bereaved at least 6 months prior to completing the questionnaires (M = 57.00 months, SD = 81.77, range = 6–685). Participants were recruited through bereavement charity mailing lists, social media advertisements and the Google content network. No upper restrictions were placed on the length of time since bereavement. Participants were included if they indicated that the deceased was a close loved one as opposed to an acquaintance or a distant friend or relative.

### Measures

#### Symptom measures

Participants completed the Prolonged Grief Disorder 13 (PG-13) scale [32] to assess PGD symptoms, and the PTSD Checklist (PCL-5, [33]) to measure PTSD symptoms. Participants who endorsed at least one item of separation distress daily (criterion B), at least five experiences of nine cognitive, emotional, and behavioural symptoms at least ‘once a day’ or ‘quite a bit’ (criterion C), and reported that these symptoms were currently resulting in significant impairment in their social, occupational, or other important areas of functioning (e.g., domestic responsibilities) (criterion D) were classified as meeting criteria for probable PGD (PGD-2009, [22]). Internal consistency of the PG-13 in this sample was excellent (α = .91).

To validate the PG-13 classification against the PCBD criteria, six additional items were added to the PG-13 that correspond to the symptoms of the PCBD criteria not represented by the PGD-2009 criteria. Two items were added to criterion B ‘how often have you been preoccupied with thoughts or memories of the deceased?’ and ‘how often have you been preoccupied with thoughts about the circumstances of the death?’ Four items were added to criterion C ‘do you have difficult recalling happy memories that involve the deceased?’, ‘do you feel bad about yourself because of things that happened in relation to the death or between you and the deceased?’, ‘do you feel that a desire to die to be with the deceased?’, ‘do you feel alone or detached from others since the death?’ The PCBD symptom ‘feeling shocked, stunned or emotionally numb’ was considered present if PGD item 5 (How often have you felt stunned, shocked or dazed by your loss?) or PGD item 11 (Do you feel emotionally numb since your loss?) were reported as present. The PCBD criterion was met if participants had been bereaved at least 12 months, endorsed at least one item of separation distress daily (criterion b), at least six of 12 symptoms of cognitive, emotional, and behavioural symptoms (criterion C), resulting in significant impairment of functioning (criterion D).

Participants who endorsed at least one re-experiencing symptom on the PCL-5 (cluster B), one avoidance behaviour (cluster C), two negative alterations in cognitions or mood (cluster D), and two hyperarousal symptoms (cluster E) as being ‘moderately present or higher’ with a current impairment in functioning as a result of these symptoms were classified as having met criteria for probable PTSD. Internal consistency of the PCL-5 in this sample was excellent (α = .94).

*Oxford Grief Study Cognitive Measures*. Comprehensive measures of loss-related memory characteristics, negative appraisals, maladaptive coping strategies, grief resilience, and social disconnection were developed from the literature on cognitive processes in PTSD [34–37] and grief [38,39], in collaboration with therapists experienced in the treatment of traumatic loss and from interviews with bereaved individuals with and without PGD [40]. Concepts relevant to development of PTSD and PGD such as qualities of memory, negative beliefs about the self, life, and grief as well as ruminating on the trauma/loss and the injustice of the event/loss [38,39] were combined with themes arising from qualitative interviews not previously described in the literature. These measures were subject to exploratory and confirmatory factor analyses and psychometric testing. All measures met the requirements of factorial validity and acceptable internal consistency, test-retest reliability and convergent, criterion and divergent validity [41–43].

***Loss-related memory characteristics (OG-M; 42)***–This 27-item questionnaire is hypothesised to act as a metric for loss integration and asks participants to rate on a 5-point scale (0 –not at all to 4 –very strongly) the extent to which each statement regarding their memories of the loss applied to them during the last month. Twenty-three items probed memory triggers and their consequences (e.g., ‘*I am reminded of the loss for no apparent reason’)*, qualities of memory (e.g., ‘*Memories of things we did together are painful’*), the poor availability of positive memories (e.g., ‘*I struggle to remember positive times without [–]’*), and the physical impact of loss-related memories (e.g., ‘*The memories of [–] ’s death make my body ache with overwhelming fatigue’)*. Four further items, taken from the Intrusions Questionnaire [35], asked about unintentional memories of the loss (frequency in the last week, distress, how much they seemed to be happening now instead of in the past, and the extent to which they felt as though they were reliving the memory). The total OG-M scale demonstrated excellent internal consistency (ω = .97) and has a unidimensional factor structure [42].

***Negative grief appraisals (OG-A)***–This 35-item questionnaire asks participants to indicate on a 7-point scale (1—totally disagree to 7—totally agree) the extent to which they agree with statements of loss-related appraisals. Items pertain to five content factors: 1. Loss of self and life (e.g. ‘*Without [–] I can never be strong again’)*, 2. Regret (*e*.*g*. *‘I blame myself for things I did or did not do when [–] was alive’*), 3. Catastrophic consequences of grief (*e*.*g*. ‘*If I start to cry I won’t be able to stop’*), 4. Loss of relationships and future (*e*.*g*. *‘I cannot maintain previous relationships without [–]’*), 5. Fear of losing connection to the deceased (*e*.*g*. *‘Letting go of my grief would mean betraying [–]’*). The total negative appraisals scale demonstrated excellent internal consistency (ω = .98).

***Unhelpful coping strategies (OG-CS)***–This 23-item questionnaire asks participants on a 5-point scale (1—never to 5 –always) to indicate how often they used particular strategies to cope with their loss that are thought to maintain grief cognitions and symptoms [39,44]. Items pertain to four content factors: 1. Avoidance (*e*.*g*., *‘I avoid places we went together’*), 2. Proximity seeking (*e*.*g*.,*’ I feel compelled to surround myself with things that they liked’*), 3. Grief rumination (*e*.*g*., *‘I dwell on moments that could have changed the outcome’*), and 4. Injustice rumination (*e*.*g*., *‘I think about the unfairness of the loss’*). The total maladaptive coping strategies scale demonstrated excellent internal consistency (ω = .96).

***Social disconnection (OG-SD; 41)***–This 15-item questionnaire asks participants to indicate on a 7-point scale (1—totally disagree to 7—totally agree) the extent to which they agree with statements about three factors of perceived disconnection from others: 1. Negative interpretation of others’ reactions (e.g. *‘Others would judge me if I were to speak openly about my grief’*), 2. Negative interpretation of social situations (e.g. *‘I can’t be myself around other people the way I used to’*), 3. Safety in solitude (e.g. *‘It is easier to be alone than to have to pretend to feel ok’*). The total social disconnection scale demonstrated excellent internal consistency (α = .94).

***Grief resilience (OG-GR)***–This 7-item questionnaire asks participants to indicate on a 7-point scale (1—totally disagree to 7—totally agree) the extent to which they agree with statements about two content domains: 1. Continuing bonds (e.g., *‘My memories of our time together give me confidence’*), 2. Self-efficacy (e.g., ‘*Even without [–]*, *I can deal with the ups and downs of life’*). The total grief resilience scale demonstrated acceptable internal consistency (α = .78).

#### Pre-loss individual differences measures

*Experiences in Close Relationships Scale Revised [ECR-S; 45].* Attachment anxiety and avoidance were measured with a validated 12-item version of the ECR-S. Participants rated their agreement with six items measuring attachment anxiety (e.g., *‘I need a lot of reassurance that I am loved by my close loved ones’*) and six items measuring attachment avoidance (e.g., *‘I do not often worry about being abandoned’*) on seven-point scales from 1 (strongly disagree) to 7 (strongly agree). The anxious attachment subscale of the ECR-S demonstrated good internal consistency (α = .80), while the avoidant attachment subscale was acceptable (α = .79).

*Dependency*. This 16-item questionnaire asks participants to indicate on a 5-point scale (1 –Not at all true of our relationship to 5 –Very true of our relationship) to the extent to which the bereaved individual had depended on the deceased both emotionally (*‘I had people other than [–] who I could confide in and share my worries with’)* and practically (*‘[–] did everything for me in our relationship’)*. Items from the healthy dependency subscale of the Relationship Profile Test were also included reflecting an individual’s ability to trust and turn to others in times of need [46]. The 6-item deceased dependency subscale demonstrated good internal consistency (α = .81), as did the 10-item healthy dependency subscale (α = .86).

### Administration procedure

Participants completed self-report symptom measures and the Oxford Grief Study measures of memory characteristics, negative appraisals, maladaptive coping strategies, social disconnection, and grief resilience as well as validated measures of attachment and dependency and psychopathology online in accordance with ethical guidelines [47]. Informed consent was obtained from participants electronically and the study was approved by the University of Oxford Medical Sciences Inter-Divisional Research Ethics Committee MS-IDREC-C1-2015-230.

### Data analysis

#### Background variables

Individual background variables (i.e., demographics, loss characteristics, and pre-loss individual differences) of potential importance to PGD and PTSD were analysed with bivariate correlations for continuous data, rank order correlations for ordered categorical data (e.g., level of education) and one-way ANOVA for non-ordered categorical data (e.g., loss of a child, partner, parent etc.). Any background variables significantly associated with continuous psychological distress variables of PGD and PTSD were retained and controlled for in the multivariate analyses.

Group comparisons were conducted with multinomial logistic regression (MNLR), an extension of a basic logistic regression, which allows more than two categories of the outcome variable. It is also considered more robust against violations of the assumptions of large and unequal sample group sizes, normally distributed errors and a linear relationship between the dependent and independent variables [48], compared with statistical techniques with similar aims (e.g. analysis of variance).

#### Group differences

According to their PG-13 [32] and PCL-5 scores [33], participants were grouped into four groups: probable PGD diagnosis only, probable PTSD diagnosis only, both PGD and PTSD, and neither PGD nor PTSD. The four groups were compared using a series of multinomial logistic regression analyses (MNLR). First, univariate analyses of group differences were conducted for each of the five cognitive behavioural measures (memory characteristics, negative appraisals, coping strategies, social disconnection, and grief resilience) to determine their relationships with the groups separately. To account for these multiple comparisons, Bonferroni alpha adjustment set the significance level for each univariate model to *p* < .01 (α/5).

To account for the influence of conceptual overlap between the cognitive mechanisms detailed in the questionnaires and the symptoms of PGD, PTSD four additional MNLR were conducted. Symptoms representing potential overlap (e.g., the avoidance item from the PGD conceptualisation) were grouped and added to each model as a predictor, leaving only the variance not shared between variables to predict group membership (see S1 Conceptual Overlap).

Next, multivariate analyses investigated the unique effects of the five cognitive predictors when entered together into a MNLR, controlling for background variables and pre-loss individual differences of importance. The cognitive variables (memory characteristics, negative appraisals, coping strategies, social disconnection, and grief resilience) were forced into the model as main effects and a backwards elimination method was used to ensure that only background predictors and pre-loss individual differences that significantly improved (*p* < .05) model fit remained in the model [49]. MNLR are flexible enough to allow the researcher to alter the reference category, allowing for all possible group comparisons, while the overall model statistics remain unchanged.

#### Exploratory analyses

To aid the interpretation of the pattern of group differences, exploratory univariate MNLRs for each subscale of the measures were computed.

## Results

### Prevalence of psychopathology

The PG-13 and PCL-5 scores indicated a probable clinical problem for 272 participants (42.0% of the sample). Prevalence of probable PTSD alone was 19.2% (N = 124), prevalence of PGD alone was 8.3% (N = 54), and prevalence of PGD+PTSD was 14.5% (N = 94). The non-clinical group (NoPGD/PTSD) represented 58.0% (N = 375) of the sample.

Of those at least 12 months post loss (N = 523), a probable diagnosis of PCBD was observed in 20.6% (N = 108) of the total sample with 7.6% (N = 40) meeting criteria for PCBD alone and 13.0% (N = 68) meeting criteria for PCBD+PTSD, overall, there was 94.1% (N = 492) agreement between PGD and PCBD criteria. For more detailed conceptualisation comparisons and multinomial logistic regression analyses using PCBD criteria please see S2. Given the high level of agreement between conceptualisations and previous research that demonstrated that those diagnosed with PGD 6–12 months after a death had higher subsequent risk of mental health problems and functional impairment [22] it was decided to that the PGD-2009 conceptualisation would be retained for subsequent analysis.

### Demographics and loss characteristics

Group demographics, loss characteristics and pre-loss individual differences variables for the No-PGD/PTSD, PTSD, PGD and PGD+PTSD groups are presented in Table 1.

**Table 1:** Group demographics, loss characteristics and previously established predictor variables for PGD, PTSD, PGD+PTSD, No-PGD/PTSD.

	No-PGD/PTSD	PTSD	PGD	PGD+PTSD
	(*n* = 375)	(*n* = 124)	(*n* = 54)	(*n* = 94)
**Demographics**				
Age in years *M (SD)*	50.28 (11.81)	47.30 (13.45)	52.79 (11.08)	47.83 (13.29)
Gender *N (%)* Female	305 (81.3)	99 (79.8)	43 (79.6)	84 (89.4)
Highest level of education *N (%)*				
No qualifications	9 (2.4)	2 (1.6)	8 (15.1)	3 (3.2)
High school education	102 (27.2)	43 (35.0)	20 (37.7)	38 (40.4)
University degree	173 (46.1)	52 (42.3)	19 (35.8)	40 (42.6)
Postgraduate degree	91 (24.3)	26 (21.1)	6 (11.3)	13 (13.8)
Ethnicity *N (%)*				
Caucasian	363 (96.8)	118 (95.9)	52 (96.3)	90 (95.7)
Not Caucasian	12 (3.2)	5 (4.1)	2 (3.7)	4 (4.3)
**Loss characteristics**				
Months since loss *M (SD)*	66.84 (88.01)	44.88 (59.87)	55.94 (107.15)	29.40 (31.23)
Who died? *N (%)*				
Spouse/Partner	146 (38.9)	41 (33.1)	20 (37.0)	32 (34.0)
Child	73 (19.5)	24 (19.4)	18 (33.3)	27 (28.7)
Sibling	20 (5.3)	12 (9.7)	2 (3.7)	5 (5.3)
Parent	105 (28.0)	38 (30.6)	12 (22.2)	26 (27.7)
Other relative	22 (5.9)	6 (4.8)	0 (0.0)	3 (3.2)
Close non-relative	9 (2.4)	3 (2.4)	2 (3.7)	1 (1.1)
Length of relationship (months)	339.70	361.30	320.77	333.08
*M (SD)*	(182.18)	(184.54)	(174.51)	(170.91)
How did they die? *N (%)*				
Non-violent	310 (82.7)	98 (79.0)	41 (75.9)	72 (76.6)
Violent (e.g., accident, homicide, suicide, drug overdose, medical negligence)	65 (17.3)	26 (21.0)	13 (24.1)	22 (23.4)
**Pre-loss individual differences**				
Anxious attachment *M (SD)*	19.19 (7.66)	22.25 (7.73)	23.00 (8.74)	23.41 (8.11)
Avoidant attachment *M (SD)*	18.08 (7.10)	21.17 (7.51)	20.94 (7.61)	23.79 (7.48)
Independence from deceased *M (SD)*	21.93 (5.58)	21.66 (5.39)	18.40 (5.95)	19.66 (6.04)
Healthy dependency *M (SD)*	33.71 (7.28)	29.91 (6.80)	30.51 (8.82)	28.14 (8.05)
**Cognitive predictors**				
Memory characteristics *M (SD)*	37.58 (18.94)	61.18 (16.64)	74.52 (15.21)	81.24 (11.88)
Negative Appraisals *M (SD)*	92.41 (31.82)	124.63 (31.13)	156.20 (37.93)	164.71 (33.91)
Coping strategies *M (SD)*	41.70 (11.01)	56.72 (14.37)	62.63 (16.65)	71.72 (14.71)
Social disconnection *M (SD)*	54.84 (20.10)	69.99 (15.62)	74.54 (16.31)	83.52 (12.17)
Grief resilience *M (SD)*	37.24 (5.99)	31.92 (6.38)	27.42 (8.45)	27.29 (7.75)

### Background variables

Zero order correlations of pre-loss individual differences, cognitive predictors and symptoms measures of PGD and PTSD are presented in Table 2. Being female was associated with significantly higher PGD (*r* = .15, *p* < .001), but not PTSD. Age was significantly related to PTSD (*r* = -.17, *p* < .001) but not PGD, with younger participants scoring higher on traumatic stress symptoms. Time since loss was significantly associated with both PGD (*r* = -.21, *p* < .001) and PTSD (*r* = -.21, *p* < .001) with those with more recent losses reporting higher psychological distress. Lower levels of education were significantly correlated with higher PGD (*r* = -.20, *p* = .001) and PTSD scores (*r* = -.10, *p* = .009). Losing the deceased by violent means (i.e., resulting from human (in)action such as suicide, homicide, accidental overdose, medical negligence) was associated with higher PGD (*r* = .13, *p* = .001) and PTSD (*r* = .12, *p* = .003) in this sample. When examining all levels of kinship to the deceased, there was a main effect of kinship *F*(5, 641) = 5.91, *p* < .001 and post hoc tests (Hochberg’s T2) revealed significantly higher PGD scores in those who had lost children compared with those who had lost a partner, parent, other close relative. There was no difference between levels of kinship for PTSD. Given their association with PGD and PTSD, age, gender, education, months since loss, violent loss and losing a child were retained as background variables of importance for the multivariate analyses.

**Table 2:** Zero order correlations of PGD, PTSD, cognitive predictors, and pre-loss individual differences.

		1.	2.	3.	4.	5.	6.	7.	8.	9.	10.	11.	12.	13.	14.	15.
1.	PGD	-														
2.	PTSD	.73***	-													
3.	Sex	.15***	.06	-												
4.	Age	.00	-.17***	.01	-											
5.	Months	-.21***	-.21***	.05	.12**	-										
6.	Mode	.13**	.11**	.09*	.05	.03	-									
7.	Childloss	.18**	.07	.12**	.19***	.06***	.46***	-								
8.	AxECR-S	.23***	.33***	-.07	-.18***	.04	-.05	-.13**	-							
9.	AvECR-S	.29***	.36***	.02	-.06	.06	-.00	-.08	.26***	-						
10.	InD	-.25***	-.11**	.09*	-.09*	-.06	.14***	.16***	-.16**	-.21***	-					
11.	HD-RPT	-.28***	-.37***	-.04	.15***	-.00	.04	.14***	-.40***	-.58***	.20***	-				
12.	OG-M	.83***	.77***	.15***	-.01	-.19***	.14***	.22***	.21***	.24***	-.22***	-.21***	-			
13.	OG-A	.78***	.71***	.03	-.05	-.17***	.13**	.18***	.32***	.40***	-.29***	-.41***	.79***	-		
14.	OG-CS	.72***	.74***	.09*	-.09*	-.21***	.15***	.19***	.27***	.28***	-.21***	-.27***	.81***	.78***	-	
15.	OG-SD	.62***	.67***	.12**	-.06	-.11**	.14**	.18***	.31***	.45***	-.15***	-.46***	.65***	.71***	.60***	-
16.	OG-GR	-.56***	-.58***	.01	.04	.09*	-.05	-.04	-.24***	-.42***	.17***	.47***	-.49***	-.64***	-.48***	-.49***

*Note*. Zero order correlations for cross-sectional data (*n* = 647). PG-13 = Prolonged grief disorder scale total score; PTSD = posttraumatic stress disorder scale total score; Months = Months since loss; LR = Length of relationship; Mode = Mode of death (Violent vs Non-Violent); Childloss = Parental relationship to deceased (Yes/No); AxECR-S = Anxious attachment style Experiences in Close Relationships–Short version; AvECR-S = Avoidant attachment style Experiences in Close Relationships–Short version; InD = Independence from deceased; HD-RPT = Healthy dependency subscale–The Relationship Profile Test; OG-M = Loss-related memory characteristics; OG-A = Negative grief-related appraisals; OG-CS = Maladaptive coping strategies; OG-SD = Social disconnection; OG-GR = Grief resilience. Point biserial correlations presented are for continuous-dichotomous variables and Phi is presented for comparisons of dichotomous variables.

Pearson’s correlation (two-tailed)

*p* < .05*

*p* < .01**

*p* < .001***.

#### Pre-loss individual differences

The measure of anxious attachment was significantly associated with both PGD (*r* = .23, *p* < .001) and PTSD (*r* = .33, *p* < .001) with a higher anxious attachment relating to higher psychological distress. Avoidant attachment showed similar results (PGD; *r* = .29, *p* < 0.001, PTSD; *r* = .36, *p* < .001). Individuals who reported higher healthy dependency (*r* = -.28, *p* < .001) and independence from the deceased (*r* = -.25, *p* < .001) had lower levels of PGD. This pattern was also reflected for PTSD (healthy dependency; *r* = -.37, *p* < .001, independence; *r* = -.11, *p* = .005). All pre-loss individual differences were retained for the multivariate analyses.

#### Cognitive predictors

All five cognitive predictor variables were significantly associated with both PGD (memory characteristics; *r* = .83, *p* < .001, appraisals; *r* = .78, *p* < .001, coping strategies; *r* = .72, *p* < .001, social disconnection; *r* = .62, *p* = .001, grief resilience; *r* = -.56, *p* < .001) and PTSD (memory characteristics; *r* = .77, *p* < .001, appraisals; *r* = .71, *p* < .001, coping strategies; *r* = .74, *p* < .001, social disconnection; *r* = .68, *p* = .001; grief resilience; *r* = -.58, *p* < .001).

### Group differences in cognitive predictors univariate analyses

After alpha correction for multiple comparisons, univariate analyses showed that each of the five cognitive measures significantly predicted variance in the dependent variable clinical diagnoses (*p* < .001): each distinguished between the non-clinical group and the PTSD, PGD, and PGD+PTSD groups in the expected directions (see Table 3). The largest ORs indicating higher scores, or in the case of grief resilience lower scores, were seen for PGD+PTSD, followed by PGD, and PTSD. Compared to the PTSD group, the PGD group had significantly higher mean scores on memory characteristics, appraisals, coping strategies, and grief resilience but did not differ on social disconnection. The PGD+PTSD group reported significantly higher mean scores on each of the five cognitive predictors compared to the PTSD group and significantly higher scores on memory characteristics, coping strategies, and social disconnection compared to the PGD group.

**Table 3:** Univariate parameter estimates of group comparisons for cognitive predictor variables.

		Reference group					
		NoPGD/PTSD		PTSD		PGD	
		B (SE)	OR (95% CI)	B (SE)	OR (95% CI)	B (SE)	OR (95% CI)
PTSD	Memory characteristics	.07 (.01)	1.07 (1.05–1.08) ***				
	Appraisals	.03 (.00)	1.03 (1.02–1.04) ***				
	Coping strategies	.09 (.01)	1.09 (1.07–1.11) ***				
	Social disconnection	.05 (.01)	1.05 (1.03–1.06) ***				
	Grief resilience	-.13 (.03)	.88 (.84 –.91) ***				
PGD	Memory characteristics	.11 (.01)	1.12 (1.10–1.15) ***	.05 (.01)	1.05 (1.03–1.07) ***		
	Appraisals	.06 (.01)	1.06 (1.05–1.07) ***	.03 (.01)	1.03 (1.02–1.04) ***		
	Coping strategies	.11 (.01)	1.12 (1.09–1.15) ***	.03 (.01)	1.03 (1.01–1.05) *		
	Social disconnection	.06 (.01)	1.06 (1.04–1.09) ***	.02 (.01)	1.02 (.99–1.04)		
	Grief resilience	-.21 (.03)	.81 (.76 –.87) ***	-.08 (.03)	.93 (.87 –.99) *		
PGD+PTSD	Memory characteristics	.15 (.01)	1.16 (1.14–1.19) ***	.09 (.01)	1.09 (1.06–1.11) ***	.04 (.01)	1.04 (1.01–1.07) **
	Appraisals	.06 (.01)	1.07 (1.06–1.08) ***	.03 (.01)	1.03 (1.02–1.04) ***	.01 (.01)	1.01 (1.00–1.02)
	Coping strategies	.15 (.01)	1.16 (1.14–1.19) ***	.06 (.01)	1.07 (1.05–1.09) ***	.04 (.01)	1.04 (1.02–1.06) **
	Social disconnection	.11 (.01)	1.11 (1.09–1.14) ***	.06 (.01)	1.07 (1.05–1.09) ***	.05 (.02)	1.05 (1.02–1.08) **
	Grief resilience	-.22 (.03)	.80 (.76 –.85) ***	-.09 (.03)	.92 (.87 –.97) **	-.01 (.03)	.99 (.93–1.05)

*Note*.

*p* < .05*

*p* < .01 **

*p <* .001***.

### Conceptual overlap

Results of the four additional analyses controlling for symptoms with conceptual overlap are the same as presented in the univariate analyses, with the exception of memory characteristics in the PGD vs PGD+PTSD comparison which did not reach significance (S1 Table A1 in S1 File).

The results for the exploratory MNLRs of the subscales of the appraisals, coping strategies, social disconnection, and grief resilience scales are shown in S3, Table A3 in S3 File. All subscales distinguished the clinical groups (PTSD, PGD, PGD+PTSD) from the non-clinical group. All clinical groups endorsed regret beliefs to a similar degree. For appraisals about loss of life and self, loss of relationships and future, and fear of losing connection, both the PGD and PGD+PTSD groups scored higher than the PTSD group. Catastrophic consequences of grief appraisals distinguished the comorbid PGD+PTSD group from the PGD alone group, and both scored higher than the PTSD group.

For coping behaviours, the PTSD and PGD groups had similar scores for avoidance and loss rumination, and both scored lower than the PGD+PTSD group. The PTSD group had significantly lower scores on proximity seeking and injustice rumination than the PGD and PGD+PTSD groups, which did not differ.

The PGD+PTSD scored higher on all subscales of the social disconnection measure (negative interpretation of others’ reactions, safety in solitude, and altered social self) than the PTSD and PGD alone groups. In addition, the PGD group scored higher than the PTSD group on the altered social self subscale.

Finally, on the grief resilience scale, the PTSD group scored higher than the PGD+PTSD group on the continuing bonds subscale, and higher than both the PGD and PGD+PTSD group on the self-efficacy measure.

### Group differences in cognitive predictors multivariate analyses

To determine the unique variance explained by each of the cognitive measures after accounting for the variance shared between them and relative contribution of background variables (i.e., age, gender, education), loss characteristics (i.e., months since loss, violent loss and losing a child), and pre-loss individual differences (i.e., attachment style and dependency), three MNLR were run using the backwards selection method. Of these variables, only months since loss (χ^2^ (3, N = 647) = 8.78, *p* = .03) significantly contributed to model fit and therefore remained in the final model. Table 4 presents the parameter estimates for the multivariate group comparisons. Every month post bereavement increased the likelihood of being in the PGD, PTSD, or non-clinical group by 1% compared with the PGD+PTSD group. No other groups differed significantly on time since loss.

**Table 4:** Multivariate parameter estimates of group comparisons for months since loss and cognitive predictor variables.

		Reference group					
		No-PGD/PTSD		PTSD		PGD	
		B (SE)	OR (95% CI)	B (SE)	OR (95% CI)	B (SE)	OR (95% CI)
PTSD	Memory characteristics	.05 (.01)	1.05 (1.03–1.07) ***				
	Appraisals	-.01 (.01)	1.00 (.98–1.01)				
	Coping strategies	.04 (.01)	1.04 (1.01–1.06) **				
	Social disconnection	.01 (.01)	1.01 (.99–1.02)				
	Grief resilience	-.10 (.02)	.90 (.87 –.94) ***				
	Months since death	-.00 (.00)	1.00 (1.00–1.00)				
PGD	Memory characteristics	.09 (.02)	1.09 (1.06–1.13) ***	.04 (.02)	1.04 (1.01–1.08) **		
	Appraisals	.02 (.01)	1.02 (1.00–1.04) *	.03 (.01)	1.03 (1.01–1.04) **		
	Coping strategies	.01 (.02)	1.01 (.97–1.04)	-.03 (.02)	.97 (.94–1.00)		
	Social disconnection	-.02 (.01)	.98 (.95–1.01)	-.03 (.01)	.97 (.95–1.01)		
	Grief resilience	-.14 (.03)	.87 (.82 –.92) ***	-.04 (.03)	.96 (.91–1.01)		
	Months since death	.00 (.00)	1.00 (1.00–1.01)	.00 (.00)	1.00 (1.00–1.01)		
PGD+PTSD	Memory characteristics	.10 (.02)	1.11 (1.07–1.15) ***	.06 (.02)	1.06 (1.03–1.09) ***	.02 (.02)	1.02 (.98–1.05)
	Appraisals	.01 (.01)	1.01 (.99–1.03)	.02 (.01)	1.02 (1.00–1.03) *	-.01 (.01)	.99 (.97–1.00)
	Coping strategies	.03 (.02)	1.04 (1.00–1.07) *	-.00 (.02)	1.00 (.97–1.04)	.03 (.02)	1.03 (1.00–1.07)
	Social disconnection	.03 (.02)	1.04 (1.01–1.07) *	.03 (.01)	1.03 (1.00–1.06) *	.05 (.02)	1.06 (1.02–1.09) **
	Grief resilience	-.12 (.03)	.89 (.84 –.94) ***	-.02 (.03)	.98 (.93–1.04)	.03 (.03)	1.03 (.97–1.09)
	Months since death	-.01 (.01)	.99 (.98–1.00) *	-.01 (.01)	.99 (.98–1.00) *	-.01 (.01)	.99 (.98–1.00) *

*Note*.

*p <* .05*

*p* < .01**

*p* < .001***.

For all three clinical groups, higher memory characteristics and lower grief resilience contributed unique variance to their differences from the non-clinical group. In addition, unhelpful coping strategies were elevated in the PTSD group, appraisals in the PGD group, and social disconnection and coping strategies in the comorbid PGD+PTSD group.

Higher memory characteristics and appraisals contributed unique variance to the distinction of the PGD and PGD+PTSD groups from the PTSD group. Elevated perceptions of social disconnection uniquely distinguished the PGD+PTSD from both the PTSD and PGD groups.

### Comparison of PGD and PCBD results

The results for the univariate analyses for PCBD criteria were largely similar (see S4, Table A4 in S4 File). A difference was the cognitive measures did not reach significance in the PCBD+PTSD versus PCBD comparison. However, social disconnection was elevated in PCBD compared to PTSD.

Similarly, for the multivariate analysis using PCBD criteria, the pattern of results is comparable to the PGD analysis but marginally weaker in magnitude, resulting in the loss of some significant findings (see S5, Table A5 in S5 File). Overall, it is to be expected that the smaller sample with a minimum time since loss set at 12 months led to loss of power and weaker results in the multivariate PCBD analysis. In fact, when excluding time frame from the PCBD criteria and re-running the analyses, the univariate results are the same as reported here, with the exception of social disconnection in PGD and PGD+PTSD comparison which approached significance (*p* = .06). In the multivariate analyses, the results are the same as presented here for PGD, with the exception of coping strategies, in the nonclinical and PCBD+PTSD comparison, appraisals in the comparison of the PTSD versus PCBD+PTSD group, and memory characteristics in the PTSD versus PCBD comparison, which did not differ.

## Discussion

This study reports the differences in cognitive predictors (i.e., memory characteristics, appraisals, coping strategies, social disconnection, grief resilience) between four groups of bereaved individuals. While almost 60% of the community sample demonstrated resilience to both PGD and PTSD, a substantial proportion of individuals reported bereavement-related mental health problems. This is in line with findings that suggest resilience to grief is shown by 45–65% of grievers [30,50]. The largest clinical group was the PTSD only group (19.2%), followed by the comorbid group PGD+PTSD (14.5%), and the PGD alone group (8.3%). These prevalence rates are somewhat higher than previous research that reports rates of PGD to between 5–10% in the general population [22,51]. One reason for this could be the number of losses by violent means in our sample, 20% had experience violent losses and the rate of PGD amongst this group was 24%. This is in line with other studies reporting higher rates of PGD in those bereaved via violent means [52,53]. In our sample, a probable diagnosis of PTSD was observed in 63.5% of individuals suffering from PGD. This rate of comorbidity is slightly higher than that reported in a sample of treatment seeking complicated grief sufferers in which 48.5% of CG sufferers also met criteria for PTSD [54]. Rates of PTSD are also broadly consistent with findings reporting up to 50% of a sample of conjugal grievers met criteria for a PTSD diagnosis in the first 2 years of loss [55], and those bereaved through illness [56]. In comparing those bereaved at least 12 months, there was 94.1% agreement between the conceptualisations of PGD and PCBD. Previous studies have reported that once present grief symptoms hardly decrease beyond 6 months post-loss and when assessed at 6 months these symptoms predict future mental and physical health problems [22,57,58]. Therefore, the main analyses used the PGD diagnostic conceptualisation as it had a more inclusive time frame.

The results provide evidence that bereaved people with PGD, PTSD and PGD+PTSD show different cognitive risk factor profiles. Individually each of the five cognitive predictors distinguished between the No-PGD/PTSD group and the clinical groups (PTSD, PGD, PGD+PTSD). Of the clinical groups, the PTSD group reported the overall the lowest levels of loss-related cognitive predictors (apart from the highest scores in grief resilience), followed next by the PGD group, with the PGD+PTSD group reporting the highest levels.

When comparing the PGD and the PTSD groups, only social disconnection did not differ between groups. This pattern of results suggests that overall, the chosen loss-related cognitive factors reflect the difficulties associated with loss responding more closely than trauma reactions. If individual subscales were considered, there were some notable exceptions (appraisals of regret, avoidance, loss rumination, negative interpretations of others’ responses, safety in solitude and continuing bonds). These processes have all been found to predict PTSD after trauma in the literature, and may also apply to loss [e.g., 59].

Interestingly, three cognitive predictors (memory characteristics, coping strategies, and social disconnection) were elevated in the comorbid PGD+PTSD class compared to the PGD alone class. This may suggest that the memory features associated with grief severity and trauma symptoms may be different in nature, leading to more diverse or intense memory characteristics. Similarly, it is understandable that individuals suffering both grief and trauma-related symptoms might engage in more frequent or varied coping strategies in an attempt to manage both conditions. When individual subscales were considered, the PGD+PTSD group scored higher on avoidance and loss rumination. In addition, when dealing with two types of unpleasant symptoms, individuals also seem to hold more negative beliefs about sharing or expressing their grief in front of others or in social situations. This in turn leads them to avoid social situations or endure them with distress. These findings are in line with previous research that highlighted psychosocial problems such a self-isolation [60] and loneliness [61] as predictors of post-loss mental health problems. Problems with social disconnection were further confirmed in the comorbid group in the multivariate analysis highlighting an important social consequence of loss and trauma that has yet to be articulated in the literature.

Similar to the univariate analysis, memory characteristics were associated with clinical problems following bereavement in the multivariate analysis. The grief groups (PGD, PGD+PTSD) reported more severe loss-related memory characteristics and negative appraisals in comparison to the PTSD group, further supporting that these characteristics are more clearly associated with grief, as opposed to trauma symptoms. These findings are supported by previous research that found negative appraisals and ‘unrealness’ of the loss (a concept linked to loss memory integration) to be linked to continuous symptoms of PGD [62,63] and PTSD [12] and membership of PGD alone and PGD+PTSD classes [21].

Some of the cognitive variables that showed group differences in the univariate, did not explain unique variance in group differences in the multivariate analysis. One example is grief resilience, which was a strong predictor of group membership in the univariate analysis; however, despite decreasing the likelihood of being in one of the clinical groups it failed to distinguish between clinical groups in the multivariate analysis. The lack of disparity between clinical groups in the multivariate analysis might suggest that grief resilience items reflect general resilience to pathology following bereavement as opposed to resilience of specific grief symptoms. These results are unable to elucidate whether lower grief resilience beliefs act a risk factor for developing a bereavement-related mental health problem or whether a reduction in grief resilience is a stress-related consequence of developing PGD, PTSD or PGD+PTSD.

Results suggested that a more recent loss increased the likelihood of being in the PGD+PTSD group compared with all of the other groups. These results are broadly in line with a recent study that found the resilient classes’ losses had occurred longer ago than the comorbid PGD+Depression class [18].

Very few studies have compared the cognitive risk profile of a resilient group with that of a group of PGD, PTSD and PGD+PTSD sufferers; one example is that of Djelantik and colleagues [15]. They compared the correlates of resilient, PGD, PGD+PTSD groups in a Dutch community sample. In comparison to the resilient class, participants in the PGD group were more likely to have lost a partner or a child, while those in the PGD+PTSD class were more likely to have lost a partner or a child, experienced bereavement via violent means, and have a lower level of education. In the present study, relationship to the deceased was associated with symptoms of PTSD and PGD; however, when included in a model with the cognitive predictors it failed to contribute to model fit and as such was removed from the model. These results suggest that cognitive variables have more to offer in explaining variance in grief and trauma symptoms following loss than background variables, loss characteristics and pre-loss individual differences. By highlighting the relative importance of modifiable cognitive predictors over and above fixed moderator variables such as relationship to the deceased or level of education, these results are encouraging for future treatment development. However, it remains possible that background variables such as gender or type of loss may act as moderators of the relationship between cognitive predictors, grief, and trauma symptoms. Future research investigating these relationships is likely to add to our understanding of ‘what works best for whom’ when developing tailored interventions.

The current study extends the literature on cognitive behavioural factors and their association to grief severity and traumatic stress symptoms in a bereaved community sample. Nonetheless, there are some limitations that are worth noting. The groups in this sample were derived by symptom criteria specified in PGD-2009 [22] and PCBD in DSM-5 [23]. However, by contrast, many of the studies cited in here used latent class analyses to derive class membership. Latent class analysis determines whether subgroups of individuals exist within the data based on similar symptom profiles. This method has been developed as an alternative to the binary diagnostic system as it allows for more categories of individuals to be represented (i.e., low, moderate, and high probability of PGD). However, this method only allows comparison of statistically extracted classes. For example, in a study of disaster bereaved individuals [16], only 3 classes were found (resilient, PGD, PGD+PTSD+Depression). The absence of a PTSD group prevented the opportunity to examine the correlates of PGD and PTSD alone classes. While latent class analysis has much to offer in terms of flexibility of modelling, our interest in this study was to investigate whether cognitive measures were able to distinguish between non-clinical responders, PGD, PTSD and how comorbid PGD+PTSD changed the profile. Given our *a priori* hypotheses, our chosen analysis was deemed the most appropriate. However, it would be of interest to determine whether these groups with probable diagnoses exist within the data when using a latent class approach and how these classes differ in their reporting of cognitive variables. The cognitive measures predicted group membership even after controlling for symptoms from the PGD and PTSD scales with overlapping content. Characterisation of cognitive factors linked to mental health symptoms allows advances in intervention strategies but is analytically difficult to parcel out. Future research may wish to restrict predictors so as to more clearly disentangle the role of overlap between cognitive behavioural factors and symptoms.

Another limitation is that our participants were predominantly Caucasian and female. Women tend to be over-represented in grief research with previous research in community samples reporting between 73.5–87% [15,64,65]. Given the lack of a significant contribution of gender in our multivariate analysis we currently have no reason to assume that associations between cognitive predictors and symptom levels are different for men and women. However, future research should aim for gender equality within samples to further confirm these assumptions. In terms of ethnic diversity within the sample our rate of white respondents (96.4%) was marginally higher than that reported by the UK census (87.1%) [66]. In light of this discrepancy, and to ensure the cultural sensitivity of these results, it will be important for future studies to investigate whether the clinical correlates reported here hold for grievers from different ethnic backgrounds and cultural contexts.

Notwithstanding these limitations, this study provides evidence of cognitive behavioural factors that are associated with PGD, PTSD, and comorbid symptom profiles. The results suggest that the grief-specific measures of memories, appraisals and coping [see also 38,39] are useful in distinguishing symptoms of PGD from PTSD following bereavement and could be useful in tailoring clinical interventions for bereaved individuals. Furthermore, this study adds to previous findings that PGD-2009 criteria [22] and PCBD [23] diagnose similar rates of people with disordered grief [67] and may represent the same diagnostic entity [68].

## Supporting information

S1 File Conceptual overlap. Univariate MNLR results controlling for conceptual overlap.
(PDF)

S2 File PCBD and PGD conceptualisation comparison. (PDF)

S3 File Subscale analyses by scale. Univariate MNLR by subscale.
(PDF)

S4 File PCBD comparison of cognitive measures. Univariate MNLR by measure.
(PDF)

S5 File PCBD comparison of cognitive measures, background variables and pre-loss individual differences. Multivariate MNLR.
(PDF)
