# Supplementary material for: Prolonged grief and posttraumatic stress disorder following the loss of a significant other: An investigation of cognitive and behavioural differences
Source: PLoS One. 2021 Apr 1;16(4):e0248852. doi: 10.1371/journal.pone.0248852 (PMC8016232; doi:10.1371/journal.pone.0248852)
Supplement: S1 File — Univariate MNLR results controlling for conceptual overlap. (PDF) [file pone.0248852.s001.pdf]

S1 Conceptual overlap

In order to investigate the role of overlapping content between the cognitive measures and the symptoms of PTSD and PGD a further four MNLR were conducted assessing the overlap of the memory characteristics, appraisals, coping strategies and social disconnection scales (see Table A1).

Analysis 1 included a mean score of items 1, 3, and 4 of the PTSD scale as a predictor alongside memory characteristics. These symptoms assess intrusive memories, reliving of the stressful event, and feeling upset when reminded of the loss and could reasonably overlap with measurement of unwanted memories of the loss, the extent to which the memory was being relived in the here and now, and fatigue in response to memories, all assessed in the memory characteristics questionnaire.

Analysis 2 investigated appraisals. Items 7, 11, and 13 from the PGD criteria assess feelings of confusion about one's role in life, difficulty moving on or making plans for the future, and a sense that life is unfulfilling or meaningless since the loss, respectively. The appraisals factors 'Loss of self and life' and 'Loss of relationships and future' characterise the specific appraisals thought to drive the feelings described in the PGD symptoms. Items 9 and 10 of the PTSD conceptualisation measure strong negative beliefs about the self, the world and others and blaming others or yourself for the loss and overlap with the appraisal's factors Loss of self and life and Regret. Mean scores were created for each symptom set (PGD and PTSD) and entered into the model alongside the appraisals measure.

Analysis 3 included item 5 from the PGD scale 'avoidance of reminders that deceased is gone' and a mean score of items 6 and 7 of the PTSD scale 'Avoidance of external reminders and 'Avoiding memories, thoughts, or feelings,' respectively. The coping strategies factor avoidance characterises these symptoms by describing specific avoidance

behaviours associated with PGD. The avoidance symptom of PGD and a mean score of the avoidance symptoms of PTSD were entered into the model together with coping strategies.

Analysis 4 included PTSD symptom 13 ‘I feel distant or cut off from other people’ as a predictor as one could argue overlap with the social disconnection scale that measures the thoughts and behaviours hypothesised to drive social disconnection.

After systematically controlling for overlapping symptoms the results of these analyses match that of the univariate analyses presented in the paper with the exception of the memory characteristics in PGD versus PGD+PTSD comparison which did not reach significance. Only the cognitive measures are presented in Table A1 for brevity.

Table A1.

Univariate parameter estimates of group comparisons for cognitive predictor variables after controlling for overlapping symptoms

		Reference group					
		NoPGD/PTSD		PTSD		PGD	
		B (SE)	OR (95% CI)	B (SE)	OR (95% CI)	B (SE)	OR (95% CI)
PTSD	Memory characteristics	.03 (.01)	1.03 (1.02 – 1.05) ***				
	Appraisals	.02 (.01)	1.02 (1.01 – 1.03) ***				
	Coping strategies	.06 (.01)	1.07 (1.04– 1.09) ***				
	Social disconnection	.02 (.01)	1.02 (1.00 – 1.03) *				
PGD	Memory characteristics	.10 (.01)	1.11 (1.08 – 1.4) ***	.07 (.01)	1.07 (1.05 – 1.10) ***		
	Appraisals	.03 (.01)	1.03 (1.02 – 1.05) ***	.01 (.01)	1.01 (1.00 – 1.03) *		
	Coping strategies	.10 (.01)	1.11 (1.08 – 1.13) ***	.04 (.01)	1.04 (1.01 – 1.07) **		
	Social disconnection	.04 (.01)	1.04 (1.01 – 1.06) **	.02 (.01)	1.02 (.99 – 1.04)		
PGD+PTSD	Memory characteristics	.12 (.01)	1.13 (1.10 –1.16) ***	.09 (.01)	1.10 (1.07 – 1.13) ***	.02 (.02)	1.02 (.99 – 1.05)
	Appraisals	.03 (.01)	1.03 (1.02 – 1.05) ***	.02 (.01)	1.02 (1.01 – 1.03) **	.00 (.01)	1.00 (.99 – 1.02)
	Coping strategies	.13 (.01)	1.14 (1.11 – 1.17) ***	.07 (.01)	1.07 (1.05 – 1.09) ***	.03 (.01)	1.03 (1.02 – 1.06) *
	Social disconnection	.06 (.01)	1.06 (1.04 – 1.09) ***	.04 (.01)	1.04 (1.02 – 1.07) ***	.03 (.01)	1.03 (1.00 – 1.06) T

Note. $p < .10^{T}$ $p < .05^{*}$ $p < .01^{**}$ $p < .001^{**}$
