# Supplementary material for: Prolonged grief and posttraumatic stress disorder following the loss of a significant other: An investigation of cognitive and behavioural differences
Source: PLoS One. 2021 Apr 1;16(4):e0248852. doi: 10.1371/journal.pone.0248852 (PMC8016232; doi:10.1371/journal.pone.0248852)
Supplement: S2 File — (PDF) [file pone.0248852.s002.pdf]

Comparison of PGD and PCBD conceptualisations

Comparison of PGD and PCBD conceptualisations are presented in Table A2. Of those at least 12 months post-loss, 13 individuals met criteria for a probable diagnosis of PGD but not PCBD (5 who were previously in the PGD only group were classified as non-clinical and 8 previously in the PGD+PTSD group were now in the PTSD only group).

Eighteen individuals gained a diagnosis of PCBD, 13 individuals previously been classified as PTSD only gained a PCBD+PTSD diagnosis and 5 previously classified as non-clinical gained a PCBD only diagnosis. Of these 18 individuals, 83.3% (N=15) did not meet the minimum symptom requirements for PGD criterion C and 38.9 % (N = 7) did not meet PGD separation distress criteria B.

Table A2.

Comparison of diagnostic conceptualisations PGD and PCBD in those bereaved > 12 months

	NoPCBD/PTSD	PTSD	PCBD only	PCBD+PTSD
NoPGD/PTSD	317	0	5	0
PTSD	0	85	0	13
PGD only	5	0	35	0
PGD+PTSD	0	8	0	55

In order to determine if there were individuals who met the minimum symptom criteria for PCBD (criterion b, c, and d) but were excluded based on time frame. PGD diagnosis and PCBD were compared for the whole sample (> 6 months). Twenty individuals met criteria for PGD but not PCBD criterion (9 who were previously in the PGD only group were classified as non-clinical and 11 individuals previously in the PGD+PTSD group were now classified as PTSD only). Twenty-one individuals met criteria for PCBD who did not

meet criteria for PGD (5 who were previously classified as non-clinical and 16 who were previously classifies as PTSD only).
