# Supplementary material for: Prolonged grief and posttraumatic stress disorder following the loss of a significant other: An investigation of cognitive and behavioural differences
Source: PLoS One. 2021 Apr 1;16(4):e0248852. doi: 10.1371/journal.pone.0248852 (PMC8016232; doi:10.1371/journal.pone.0248852)
Supplement: S3 File — Univariate MNLR by subscale. (PDF) [file pone.0248852.s003.pdf]

S3 Subscale analyses by measure

The univariate multinomial logistic regression analyses for each cognitive measures' subscales are presented in Table A3. Significance levels are reported alone for simplicity with asterisks indicating significantly lower scores in the reference groups.

Table A3.

Univariate subscale analyses of cognitive predictors

		Reference Group		
**Scale**		Non-clinical	PTSD	PGD
**Appraisals**				
Catastrophic Consequences of Grief	PTSD	***		
	PGD	***	*	
	PGD+PTSD	***	***	**
Regret	PTSD	***		
	PGD	**	n.s.	
	PGD+PTSD	***	n.s.	n.s.
Fear of losing connection	PTSD	***		
	PGD	***	***	
	PGD+PTSD	***	***	n.s.
Loss of Life And Self	PTSD	***		
	PGD	***	***	
	PGD+PTSD	***	***	n.s.
Loss of Relationships And Future	PTSD	***		
	PGD	***	***	
	PGD+PTSD	***	***	n.s.
**Coping strategies**		Non-clinical	PTSD	PGD
Avoidance	PTSD	***		
	PGD	***	n.s.	
	PGD+PTSD	***	***	***
Proximity Seeking	PTSD	***		
	PGD	***	***	
	PGD+PTSD	***	***	n.s.
Loss rumination	PTSD	***		
	PGD	***	n.s.	
	PGD+PTSD	***	***	**
Injustice rumination	PTSD	***		
	PGD	***	**	
	PGD+PTSD	***	***	n.s

**Social disconnection**		Non-clinical	PTSD	PGD
Negative interpretation of others' reactions	PTSD	***		
	PGD	***	n.s.	
	PGD+PTSD	***	**	**
Safety in solitude	PTSD	***		
	PGD	***	n.s.	
	PGD+PTSD	***	***	*
Altered social self	PTSD	***		
	PGD	***	*	
	PGD+PTSD	***	***	**
**Grief Resilience**		Non-clinical	PTSD	PGD
Continuing bonds	PTSD	***		
	PGD	***	n.s.	
	PGD+PTSD	***	**	n.s
Self-efficacy	PTSD	***		
	PGD	***	***	
	PGD+PTSD	***	***	n.s

Note. $p < .05^{*}$ $p < .01$ $^{**}p < .001^{***}$
