# Supplementary material for: Prolonged grief and posttraumatic stress disorder following the loss of a significant other: An investigation of cognitive and behavioural differences
Source: PLoS One. 2021 Apr 1;16(4):e0248852. doi: 10.1371/journal.pone.0248852 (PMC8016232; doi:10.1371/journal.pone.0248852)
Supplement: S4 File — Univariate MNLR by measure. (PDF) [file pone.0248852.s004.pdf]

S4 Group differences in cognitive predictors univariate analyses

After alpha correction for multiple comparisons, all five predictors significantly predicted variance in the dependent variable clinical diagnoses ($p < .001$). Results of univariate group differences in cognitive predictors are presented in Table A3. All five cognitive predictor variables distinguished between the non-clinical group and the PTSD, PCBD, and PCBD+PTSD groups in the expected directions. The largest ORs indicating higher scores, or in the case of grief resilience lower scores, were seen in the PCBD+PTSD comparison, followed by the PCBD, and the PTSD. Compared to the PTSD group, the PCBD group and the PCBD+PTSD group reported significantly higher mean scores on all five cognitive predictors. Memory characteristics and coping strategies were significantly elevated in the comorbid PCBD+PTSD group compared to the PCBD only group.

Table A4.

Univariate parameter estimates of group comparisons for cognitive predictor variables

		Reference group					
		No-PCBD/PTSD		PTSD		PCBD	
		B (SE)	OR (95% CI)	B (SE)	OR (95% CI)	B (SE)	OR (95% CI)
PTSD	Memory characteristics	.06 (.01)	1.07 (1.05 – 1.08) ***				
	Appraisals	.03 (.00)	1.03 (1.02 – 1.04) ***				
	Coping strategies	.08 (.01)	1.09 (1.07– 1.11) ***				
	Social disconnection	.04 (.01)	1.05 (1.02 – 1.07) ***				
	Grief resilience	-.13 (.02)	.88 (.84 – .91) ***				
PCBD	Memory characteristics	.10 (.01)	1.10 (1.08 – 1.13) ***	.04 (.01)	1.04 (1.01 – 1.06) **		
	Appraisals	.06 (.01)	1.06 (1.05 – 1.07) ***	.03 (.01)	1.03 (1.02 – 1.04) ***		
	Coping strategies	.12 (.01)	1.12 (1.09 – 1.15) ***	.03 (.01)	1.03 (1.01 – 1.06) **		
	Social disconnection	.10 (.03)	1.11 (1.05 – 1.16) ***	.06 (.03)	1.06 (1.01 – 1.11) *		
	Grief resilience	-.22 (.03)	.80 (.76 – .84) ***	-.09 (.03)	.91 (.87 – .96) ***		
PCBD+PTSD	Memory characteristics	.12 (.01)	1.13 (1.10 –1.16) ***	.06 (.01)	1.06 (1.04 – 1.08) ***	.02 (.01)	1.02 (1.00 – 1.05) T
	Appraisals	.06 (.01)	1.06 (1.05 – 1.08) ***	.03 (.01)	1.03 (1.02 – 1.04) ***	.00 (.01)	1.00 (.99 – 1.02)
	Coping strategies	.14 (.01)	1.15 (1.12 – 1.18) ***	.05 (.01)	1.05 (1.03 – 1.08) ***	.02 (.01)	1.02 (1.00 – 1.05)
	Social disconnection	.12 (.02)	1.12 (1.08 – 1.17) ***	.07 (.02)	1.08 (1.03 – 1.13) **	.02 (.03)	1.02 (.96 – 1.07)
	Grief resilience	-.23 (.02)	.79 (.76 – .83) ***	-.10 (.02)	.90 (.86 – .94) ***	-.01 (.03)	.99 (.94 – 1.04)

Note. $p < .10^{T}$ $p < .05^{*}$ $p < .01$ $^{**}p < .001^{***}$
