# Supplementary material for: Prolonged grief and posttraumatic stress disorder following the loss of a significant other: An investigation of cognitive and behavioural differences
Source: PLoS One. 2021 Apr 1;16(4):e0248852. doi: 10.1371/journal.pone.0248852 (PMC8016232; doi:10.1371/journal.pone.0248852)
Supplement: S5 File — Multivariate MNLR. (PDF) [file pone.0248852.s005.pdf]

S5 Group differences in cognitive predictors multivariate analyses

To determine the unique variance explained by the cognitive predictor variables after accounting for the variance shared between them and examining the relative contribution of background variables (i.e. age, gender, education), loss characteristics (i.e. months since loss, violent loss, and losing a child), and pre-loss individual differences (i.e. attachment style and dependency), three MNLR were run using the backwards selection method. Losing a child (χ^2 (3, N = 523) = 11.67, p = .01) and dependency on the deceased (χ^2 (3, N = 523) = 9.94, p = .02) were retained in the model. Table A5 presents the parameter estimates for group comparisons.

Compared to the non-clinical group, the PTSD group reported significantly higher mean level memory characteristics, coping strategies, independence from the deceased, and significantly lower grief resilience. The PTSD group were also more likely to have lost a child. The PCBD group had significantly higher scores on memory characteristics, social disconnection, and lower grief resilience. Memory characteristics, social disconnection and low grief resilience also significantly predicted membership of the comorbid PGD+PTSD group, while coping strategies was significant at trend level (p = .09).

Compared to the PTSD group, the PCBD group reported higher appraisals (p = .07), social disconnection (p = .07) and lower grief resilience (p = .06) all at trend level and significantly lower independence from the deceased, while the PGD+PTSD group had significantly higher social disconnection and lower grief resilience at trend level (p = .06). No cognitive predictors contributed significant variance in the PCBD versus PCBD+PTSD comparison.

Table A5.

Parameter estimates of multivariate group comparisons for childloss, independence from the deceased and cognitive predictor variables

		Reference group					
		No-PCBD/PTSD		PTSD		PCBD	
		B (SE)	OR (95% CI)	B (SE)	OR (95% CI)	B (SE)	OR (95% CI)
PTSD	Memory characteristics	.05 (.01)	1.05 (1.03 – 1.07) ***				
	Appraisals	-.00 (.01)	1.00 (.98 – 1.01)				
	Coping strategies	.04 (.02)	1.04 (1.01 – 1.07) *				
	Social disconnection	.01 (.01)	1.01 (.99 – 1.03)				
	Grief resilience	-.10 (.03)	.91 (.87 – .95) ***				
	Independence from deceased	.07 (.03)	1.07 (1.01 – 1.13) *				
	Child loss = 0	1.23 (.38)	3.41 (1.62 – 7.14) ***				
PCBD	Memory characteristics	.05 (.02)	1.06 (1.02 – 1.09) **	.00 (.02)	1.00 (.97 – 1.04)		
	Appraisals	.01 (.01)	1.01 (.99 – 1.03)	.02 (.01)	1.02 (1.00 – 1.04) ^{T}		
	Coping strategies	.01 (.02)	1.01 (.97 – 1.06)	-.03 (.02)	.97 (.94 – 1.01)		
	Social disconnection	.04 (.02)	1.04 (1.00 – 1.08) *	.03 (.02)	1.03 (1.00 – 1.07) ^{T}		
	Grief resilience	-.16 (.04)	.85 (.80 – .92) ***	-.06 (.03)	.94 (.88 – 1.00) ^{T}		
	Independence from deceased	-.04 (.04)	.96 (.88 – 1.04)	-.11 (.04)	.90 (.83 - .97) **		
	Child loss = 0	.59 (.53)	1.80 (.64 – 5.08)	-.64 (.52)	.53 (.19 – 1.46)		
PCBD+PTSD	Memory characteristics	.07 (.02)	1.07 (1.04 – 1.11) ***	.02 (.02)	1.02 (.99 – 1.06)	.02 (.02)	1.02 (.98 – 1.06)
	Appraisals	.01 (.01)	1.01 (.99 – 1.02)	.01 (.01)	1.01 (.99 – 1.03)	-.01 (.01)	.99 (.97 – 1.01)
	Coping strategies	.03 (.02)	1.03 (1.00 – 1.08) ^{T}	-.00 (.02)	1.00 (.96 – 1.03)	.02 (.02)	1.02 (.98 – 1.06)
	Social disconnection	.05 (.02)	1.06 (1.02 – 1.09) **	.05 (.02)	1.05 (1.02 – 1.08) **	.01 (.02)	1.01 (.98 – 1.05)
	Grief resilience	-.15 (.03)	.86 (.81 – .92) ***	-.06 (.03)	.95 (.89 – 1.00) ^{T}	.01 (.03)	1.01 (.95 – 1.07)
	Independence from deceased	.00 (.04)	1.00 (.93 – 1.08)	-.06 (.04)	.94 (.87 – 1.01) ^{T}	.05 (.04)	1.05 (.97 – 1.13)
	Child loss = 0	.77 (.47)	2.16 (.86 – 5.38)	-.46 (.44)	.63 (.27 – 1.51)	.18 (.49)	1.20 (.46 – 3.16)

Note. $p < .10^{T}$ $p < .05^{*}$ $p < .01^{**}$ $p < .001$

Comparison of PGD and PCBD

Contrary to the PGD analysis, no background variables were retained in the model, memory characteristics did not distinguish between the clinical groups (PTSD, PCBD, PCBD+PTSD), social disconnection did not differ between the grief groups (PCBD vs PCBD+PTSD), and appraisals did not reach significance in the comparison of PCBD with the non-clinical group or the PTSD with PCBD+PTSD group. However, social disconnection was significantly elevated in the PCBD alone group (vs non-clinical).
